# Posttraumatic stress disorder is associated with Alzheimer’s disease–relevant molecular remodeling in the amygdala of older Veterans

**DOI:** 10.21203/rs.3.rs-9235023/v1

**Published:** 2026-03-30

**Authors:** Macy A. Seijo, Pheven A. Yohannes, Amaya L. Rogers, Jose Carlos Gonzalez, Anisha Banerjee, Hunter Kelley, Meghan Pierce, Joseph A. McQuail, Caesar M. Hernandez

**Affiliations:** Department of Medicine, Division of Gerontology, Geriatrics, and Palliative Care, The University of Alabama at Birmingham, Birmingham, AL; Department of Medicine, Division of Gerontology, Geriatrics, and Palliative Care, The University of Alabama at Birmingham, Birmingham, AL; Department of Medicine, Division of Gerontology, Geriatrics, and Palliative Care, The University of Alabama at Birmingham, Birmingham, AL; Department of Medicine, Division of Gerontology, Geriatrics, and Palliative Care, The University of Alabama at Birmingham, Birmingham, AL; Department of Medicine, Division of Gerontology, Geriatrics, and Palliative Care, The University of Alabama at Birmingham, Birmingham, AL; National Center for PTSD Brain Bank, Department of Veterans Affairs Boston Healthcare System.; National Center for PTSD Brain Bank, Department of Veterans Affairs Boston Healthcare System.; Department of Pharmacology, Physiology, and Neuroscience, University of South Carolina School of Medicine, Columbia, SC, USA; Department of Medicine, Division of Gerontology, Geriatrics, and Palliative Care, The University of Alabama at Birmingham, Birmingham, AL

**Keywords:** Posttraumatic Stress Disorder, Aging, Amygdala, Alzheimer’s disease

## Abstract

Posttraumatic stress disorder (PTSD) has been associated with accelerated cognitive aging and increased risk for Alzheimer’s disease (AD) and related dementias (ADRD), yet the neural substrates linking trauma-related psychiatric illness to late-life neurodegenerative vulnerability remain poorly defined. The amygdala plays a central role in threat processing and emotional memory and exhibits persistent hyperactivity in PTSD, but its molecular and pathological state in aging individuals with PTSD has not been systematically examined. Postmortem amygdala tissue from older adult donors (≥ 70 years) with lifetime PTSD (n = 5) and age-matched controls (n = 5) was obtained from the National PTSD Brain Bank. A multimodal analysis was performed integrating immunohistochemical quantification of β-amyloid and phosphorylated tau pathology, targeted transcriptional profiling of AD–related genes, gene network analysis, and protein quantification of pathological, inflammatory, and synaptic markers. PTSD cases showed enrichment of combined tau–amyloid pathology within the amygdala and significantly greater β-amyloid burden. Targeted transcriptomic profiling identified coordinated upregulation of AD–related genes involved in amyloid processing, lipid metabolism, proteostasis, and inflammatory signaling. Network analysis revealed an APP-centered molecular architecture with APOE, MAPT, and CLU functioning as highly connected secondary hubs. Protein analyses demonstrated increased amyloid-β and pTau231 abundance, selective markers of gliosis, and synaptic alterations characterized by elevated excitatory receptor expression and reduced inhibitory GABABR1a. Older adults with PTSD exhibit convergent evidence of AD–relevant molecular and pathological remodeling in the amygdala. These findings suggest that chronic trauma-related circuit dysregulation may intersect with aging-associated inflammatory and synaptic processes, creating a biological environment permissive for neurodegenerative vulnerability in emotionally salient brain circuits.

## 1.0 Introduction

Posttraumatic stress disorder (PTSD) is a prevalent condition among Veterans and is associated with substantial long-term neuropsychiatric morbidity. Beyond its well-established link to suicide risk^1–3^, PTSD has increasingly been associated with accelerated cognitive aging and elevated risk for Alzheimer’s disease (AD) and related dementias (ADRD)^4–6^. Importantly, this increased dementia risk is observed even in the absence of traumatic brain injury^4^, suggesting that psychosocial trauma and chronic stress physiology may contribute directly to age-related neurobiological vulnerability. Epidemiologic studies further indicate that Veterans with PTSD experience steeper cognitive decline than age-matched controls^7^, with impairments reported across episodic memory, executive function, and attention^8^. These deficits can reach magnitudes comparable to those observed in mild cognitive impairment (MCI) in cognitively normal older adults^9^, raising the possibility that PTSD may accelerate trajectories toward neurodegenerative disease.

Converging biomarker and neuroimaging findings support this possibility. Veterans with PTSD exhibit increased incidence of AD relative to those without PTSD^4,5^, and PET imaging studies demonstrate greater tau accumulation that correlates with amyloid burden^10,11^. Such findings suggest that trauma-related psychiatric illness may interact with early AD-relevant molecular processes. However, recent PET imaging studies examining AD-related pathology in Veterans with PTSD have yielded equivocal or negative associations^12,13^. Importantly, these studies relied on global brain measures or broad regions of interest. Given the small size of the amygdala and the limited spatial resolution of PET, such approaches may lack sensitivity to detect region-specific pathological changes. This limitation highlights the need for more targeted, molecular-level analyses capable of quantifying features that are not accessible in vivo through PET imaging.

The amygdala represents a compelling candidate region linking PTSD to age-related pathology. The amygdala plays a central role in threat detection, emotional salience, fear learning, and emotional flexibility such as extinction learning and memory^14–17^. Functional imaging studies consistently demonstrate heightened amygdala activity during threat processing in individuals with PTSD, reflecting exaggerated emotional responsivity and persistent fear signaling^14–18^. Chronic stress and trauma exposure are thought to bias amygdala circuits toward hyperexcitable states that impair adaptive regulation of emotional memories and threat responses^14–17^. Notably, dysfunction of these same emotional memory circuits has been implicated in both aging-related cognitive decline and early AD pathology.

Inflammatory and metabolic processes associated with aging and neurodegeneration may further intersect with amygdala dysfunction. Neuroinflammation is a key contributor to AD pathogenesis^19–24^ and can directly enhance amygdala responsivity to threat-related stimuli^21,22^. Aging itself is accompanied by a progressive increase in inflammatory signaling^23–33^, which may lower the threshold for stress-related circuit instability. Consistent with this idea, preclinical models of AD demonstrate inflammatory remodeling and synaptic dysfunction within corticolimbic circuits, including the basolateral amygdala, prior to widespread neurodegeneration^34–37^.

Despite strong epidemiologic links between PTSD and dementia risk, relatively little is known about the molecular and pathological state of the human amygdala in older adults with PTSD. Addressing this gap is critical for understanding how trauma-related psychiatric illness may shape brain aging trajectories. To address this question, we examined postmortem amygdala tissue from older adults with and without PTSD obtained from the National PTSD Brain Bank. Using a multimodal approach integrating immunohistochemical pathology, targeted gene expression profiling, network analysis, and protein quantification, we tested the hypothesis that PTSD is associated with AD–relevant molecular remodeling within the aging amygdala.

## 2.0 Materials and Methods

### 2.1 Human Brain Tissue and Cohort Characteristics.

Fresh-frozen and formalin-fixed human amygdala tissue was obtained from the National PTSD Brain Bank (NPBB) at the VA Boston Healthcare System. All procedures were approved by the appropriate Institutional Review Boards, and written informed consent for brain donation was obtained from donors or their next of kin.

History of posttraumatic stress disorder (PTSD) and demographic information were obtained through comprehensive medical record review and structured interviews conducted with the next of kin. Trained clinicians administered standardized interviews to collect detailed health, military service, and psychiatric history. PTSD diagnoses were determined by consensus among three independent clinicians who rated the probability of lifetime PTSD on a 5-point scale ranging from 1 (“PTSD Highly Improbable”) to 5 (“PTSD Definite”). Cases classified as PTSD met consensus criteria consistent with structured clinical assessment and record review. Control donors had no documented history of PTSD or other major neuropsychiatric disorders.

Ten older adult male donors (≥ 70 years) were included and grouped as Control (n = 5) or PTSD (n = 5). Individuals with a diagnosed primary neurodegenerative disorder at the time of death were excluded. Clinical variables including age, postmortem interval (PMI), and reported history of traumatic brain injury (TBI) are summarized in Tables 1 and 2.

### 2.2 Neuropathological Processing and Tissue Preparation.

Whole brains were received fresh on wet ice. Upon arrival, brains were weighed, inspected for gross abnormalities, and photographed. A dorsoventral sagittal cut through the corpus callosum separated hemispheres.

One hemisphere was immersion-fixed in ice-cold 4% paraformaldehyde-lysine-periodate (PLP; pH 7.4) for at least two weeks prior to neuropathological evaluation. The contralateral hemisphere was processed for fresh dissection and flash-freezing. Prior to coronal sectioning, samples were collected for quality control, respectively.

The fresh hemisphere was sectioned into 0.5 cm coronal slabs at standardized anatomical landmarks and stored at − 80°C. Fixed tissue was processed into formalin-fixed paraffin-embedded (FFPE) blocks following dehydration and paraffin embedding using standard neuropathology protocols. All cases underwent formal neuropathological review and consensus diagnosis prior to release for research use.

The FFPE block corresponding to the amygdala with entorhinal cortex was identified based on gross anatomical photographs and neuropathologist confirmation. For molecular studies, frozen amygdala tissue was dissected from coronal slabs on dry ice using a Dremel-based microdissection approach with parallel neuropathological confirmation of anatomical boundaries.

Dissected frozen amygdala blocks were embedded in optimal cutting temperature (O.C.T.) compound on crushed dry ice and stored at − 80°C. Frozen blocks were sectioned at 50 μm, and the entorhinal cortex was carefully removed using a sterile razor blade to isolate amygdala-only tissue. Separate blades were used for each case to prevent cross-contamination. Ten amygdala-only frozen sections were obtained per case for downstream analyses.

### 2.3 Immunohistochemistry and Pathology.

Immunohistochemical staining was performed on 10 μm FFPE sections of amygdala tissue using a Leica BOND-III automated slide stainer (Leica Biosystems) with Leica BOND system reagents according to validated laboratory standard operating procedures. Slides underwent automated on-board deparaffinization followed by heat-induced epitope retrieval. β-Amyloid (Aβ) immunostaining was performed using monoclonal antibody clone mOC98 (Abcam, ab201061) at a working dilution of 1:6000 following antigen retrieval with Bond Epitope Retrieval Solution 1 (ER1, pH 6.0). Phosphorylated tau was detected using the AT8 monoclonal antibody (Thermo Fisher Scientific, MN1020), recognizing phospho-Tau Ser202/Thr205, at a dilution of 1:200 with antigen retrieval using Bond Epitope Retrieval Solution 2 (ER2, pH 9.0).

Primary antibody incubation and signal detection were automated using the Leica BOND Polymer Refine Detection Kit (DS9800) with 3,3′-diaminobenzidine (DAB) chromogen. Slides were counterstained with hematoxylin, dehydrated, and coverslipped using standard protocols. All staining runs included appropriate positive and negative controls. Immunoreactivity was visualized under brightfield microscopy, and images were captured using standardized exposure settings for subsequent quantitative analysis.

Whole-slide brightfield images of DAB-stained sections were acquired following immunohistochemical processing and exported for digital analysis. Quantification of β-amyloid and AT8 immunoreactivity was performed using QuPath (version 0.6.0), an open-source digital pathology software platform. For each case, the amygdala was manually delineated as a region of interest (ROI) based on established anatomical boundaries to ensure accurate exclusion of adjacent entorhinal cortex and surrounding structures. Analyses were restricted to the amygdala ROI to maintain anatomical specificity across all samples.

Images were analyzed using the Brightfield (H-DAB) image type setting. Immunoreactive signal was quantified using the Pixel Classification Thresholder applied to the DAB channel. A standardized thresholding approach was used for all cases, with image resolution set at 0.55 μm per pixel (high resolution), a DAB threshold value of 0.15, and sigma smoothing set to 2. Threshold parameters were defined prior to group comparisons and applied uniformly across all samples to minimize analytic bias. The primary outcome measure was the percentage of positive DAB pixels within the amygdala ROI, reflecting the proportion of tissue area demonstrating immunoreactivity above threshold. All image analyses were conducted blinded to diagnostic group.

### 2.4 Isolation of RNA and Protein from Human Amygdala.

To assess whether molecular pathways associated with Alzheimer’s disease (AD) pathology were altered in the amygdala of older adults with PTSD, total RNA and protein were isolated from the same tissue samples to enable direct cross-modal comparisons. Fresh-frozen amygdala tissue was processed using the PARIS^™^ Kit (ThermoFisher Scientific; AM1921), following the manufacturer’s protocol. This approach allows sequential extraction of high-quality RNA and protein from a single sample, minimizing variability arising from tissue heterogeneity. RNA and protein fractions were stored at − 80°C until downstream processing.

### 2.5 cDNA Synthesis and Alzheimer’s Disease Gene Expression Profiling.

Total RNA concentration and purity were assessed using a Thermo Scientific NanoDrop One Microvolume Spectrophotometer, and all samples met quality criteria for downstream qPCR analysis. First-strand cDNA was synthesized using Qiagen’s RT^^2^^ First Strand cDNA Synthesis Kit (Cat. No. 330421), which includes a genomic DNA elimination step via DNase incubation to minimize contamination.

Gene expression profiling was performed using RT^^2^^ Profiler^™^ PCR Array Human AD panels (Qiagen; Cat. No. 330231), which interrogate curated AD-related genes spanning amyloid processing, tau pathology, synaptic function, neuroinflammation, mitochondrial biology, and cellular stress pathways. cDNA was combined with RT^^2^^ SYBR^®^ Green ROX qPCR Mastermix (Qiagen; Cat. No. 330523), and samples were loaded onto 384-well RT^^2^^ Profiler PCR Array plates according to the manufacturer’s instructions.

Quantitative PCR was conducted using a QuantStudio 6 with cycling parameters specified in the RT^^2^^ Profiler PCR Array Handbook (Qiagen), with fluorescence acquisition during the annealing/extension phase. All internal array controls passed quality thresholds, including genomic DNA controls, reverse transcription controls, and positive PCR controls.

### 2.6 Protein Quantification and Simple Western (JESS) Analysis.

Protein concentrations were determined using a bicinchoninic acid (BCA) assay prior to immunodetection. Protein expression analyses were performed using the Simple Western^™^ JESS platform (Bio-Techne), following standardized laboratory protocols optimized for human brain tissue.

Protein samples were prepared using Bio-Techne reagents and denatured according to antibody-specific conditions determined during prior optimization. Samples were separated using the 12–230 kDa Fluorescence Separation Module (Cat. No. SM-FL001). All antibodies were validated and optimized for JESS analysis, with details regarding antibody source, catalog number, dilution, and heating conditions provided in Table 3.

To control for total protein loading and inter-sample variability, all targets were normalized using the Protein Normalization Module (Bio-Techne; DM-PN02). Targets were grouped a priori into biologically relevant categories, including AD-related pathology, gliosis and inflammation, excitatory markers, and inhibitory markers, to facilitate network-level interpretation.

### 2.7 Statistical Design.

#### 2.7.1 General statistical approach.

Unless otherwise noted, all statistical analyses were performed in SPPSS28 v280.0.0(190). For gene expression and protein data, independent samples t-tests were used as the primary statistical tests within predefined biological groupings. In all analyses, the alpha (α) was set to 0.05. When there were significant effects, the effect sizes were reported as Cohen’s d (d) or partial eta squared (η_p_^2^) for ANOVAs. Effect sizes were reported as Cohen’s d and partial η^^2^^ and interpreted using conventional benchmarks^38,39^, with values of approximately 0.2,0.5, > 0.8 for Cohen’s d and 0.01, 0.06, and 0.14 for η_p_^2^representing small, moderate, and large effects, respectively. For brevity, null effects were reported as consolidated t-statistics, p-values, and only the lowest and highest values for each were given. All figures were generated in GraphPad Prism v10.4.2(633). All data generated or analyzed during this study are included in this published article. Data are presented as mean ± SEM unless otherwise stated.

#### 2.7.2 Neuropathological Group Comparisons.

To evaluate AD–related pathology in the amygdala, we first compared the proportion of cases exhibiting both AT8-positive phosphorylated tau neurofibrillary tangles and β-amyloid positivity between PTSD and control groups. Proportions were calculated with corresponding 95% confidence intervals to characterize the precision of group-level estimates.

Because formal Braak staging and ABC scoring were not available for these cases, we operationalized a pathology index to capture cooccurrence of tau and amyloid pathology within the amygdala. Each case was assigned a score of 0 if no AT8 or amyloid positivity was detected, a score of 1 if either AT8 or amyloid positivity was present, and a score of 2 if both pathologies were present within the amygdala region of interest. Given the ordinal and non-normally distributed nature of this index and the small sample size, group differences were evaluated using a Mann–Whitney U test.

To further quantify whether PTSD was associated with increased likelihood of combined tau and amyloid pathology, odds ratios and risk ratios were calculated based on the presence of dual positivity. Crosstabulation analyses were performed in SPSS, and statistical significance of association was determined using Fisher’s exact test derived from Chi-square contingency analysis, appropriate for small cell counts.

Finally, continuous measures of pathology burden were evaluated by comparing the percentage of DAB-positive pixels within the amygdala ROI for AT8 and β-amyloid between groups using independent-samples t-tests. Assumptions of homogeneity of variance were assessed using Levene’s test, and Welch’s correction was applied when variances were unequal. To determine whether observed group differences were influenced by TBI history, markers showing group effects were subsequently evaluated using hierarchical linear regression models, with diagnostic group entered in Model 1 and TBI status added as a covariate in Model 2.

#### 2.7.3 RT-qPCR Gene Expression Data Processing.

qPCR data were analyzed using the Applied Biosystems^™^ Relative Quantification (RQ) App powered by ThermoFisher Cloud (version 2021.1.1-Q1–21-build11). Global normalization across all plates was applied using the platform’s recommended algorithms. Only genes passing quality control thresholds were included in downstream analyses.

Prior to statistical analysis, gene-level outlier detection was performed in SPSS for each target. Outliers were identified and removed on a per-gene basis to minimize undue influence of non-representative observations on group comparisons. To test our a *priori* directed hypothesis that AD-related genes are upregulated in the amygdala of older adults with PTSD, independent-samples t-tests were conducted for each of the 84 AD-related gene targets included on the RT^2^ Profiler PCR Array, comparing PTSD and age-matched non-PTSD control groups. This testing strategy was specified prior to analysis and applied uniformly across all datasets. All statistical results are provided in **Table S1**.

To ensure valid inference under violations of distributional assumptions, homogeneity of variance for all independent-samples t-tests was formally evaluated using Levene’s Test for Equality of Variances. In cases where Levene’s test indicated significantly unequal variances between groups (p < 0.05), the variance-corrected Welch’s t-test was applied and the corresponding adjusted p-value was used for statistical inference. When homogeneity of variance was satisfied, the standard pooled-variance t-test was retained. This procedure was applied consistently across all molecular analyses.

To further control for false positives arising from parallel hypothesis testing, multiple-comparison correction was performed using the Benjamini–Hochberg false discovery rate (FDR) procedure. An FDR threshold of q = 0.2 was selected based on the number of tests, the correlated structure of AD–related gene expression, and empirical inspection of the discovery curve, which showed a clear inflection point near q ≈ 0.2 (**Figure S1A**). This threshold balances sensitivity to detect directionally consistent, biologically meaningful effects with explicit control of the expected false discovery proportion. More stringent thresholds (e.g., q ≤ 0.05) were retained for high-confidence findings. All nominal p-values and FDR-adjusted results across a range of q values are provided in **Table S1**.

#### 2.7.4 Network Analysis.

All genes passing the selected FDR threshold were imported into Cytoscape (Version 3.10.3) to construct gene–gene interaction networks and facilitate pathway-level interpretation. Because the differential expression analysis had already identified significant genes, principal component analysis (PCA) was performed in SPSS using only these FDR-passing genes to reduce dimensionality and generate regression scores representing coordinated transcriptional modules. This approach enabled regression analyses controlling for TBI history at the systems level rather than requiring separate regression models for each individual gene. Missing values were replaced with group means to allow PCA estimation. Because differential expression had already been determined prior to dimensionality reduction, this procedure did not alter between-group fold-change relationships for individual genes. Importantly, PCA regression scores reflect coordinated multigene expression patterns within each sample rather than individual gene values, ensuring that dimensionality reduction captured global transcriptional structure without inflating group differences.

Regression scores derived from the PCA components were subsequently evaluated for group differences using multivariate analysis of variance (MANOVA) to determine whether the transcriptional modules identified by dimensionality reduction were collectively altered between groups. This analysis tested whether component-level gene expression patterns separated PTSD and control cases, providing confirmation that the coordinated gene modules identified by PCA were differentially expressed at the systems level. Demonstrating group separation at the module level ensured that the PCA-derived gene sets represented meaningful biological patterns suitable for integration into the Cytoscape network analyses. PCA regression scores for each retained component were entered into hierarchical linear regression models. Model 1 tested group differences in component scores (Control vs PTSD), and Model 2 added TBI status as a covariate to determine whether group-level transcriptional module differences remained stable after accounting for TBI history.

Networks were visualized in Cytoscape using an edge-weighted, spring-embedded layout, and topological properties were analyzed using the NetworkAnalyzer tool to quantify network architecture, including node degree and connectivity patterns. Complete statistics for network analyses are provided in **Tables S2** and **S3**. Spearman correlation matrices were generated separately within each experimental group and imported into Cytoscape to map correlation structure onto the validated gene interaction network. Outliers identified during preliminary analyses were excluded from statistical comparisons of gene expression but retained for correlation-based network analyses to preserve variance, as Spearman correlations reduce sensitivity to extreme values. Each gene was represented as a node, and gene–gene relationships were represented as edges.

Nodes were encoded with multiple attributes to integrate transcriptional and network-level information. Node color reflected fold change in gene expression between groups (red = increased expression in PTSD; blue = decreased expression), node border color indicated PCA component membership (gray = component 1; black = component 2), and node size corresponded to PCA loading magnitude, such that larger nodes reflected stronger component loadings. Edges were encoded to represent several statistical properties of gene–gene relationships. Edge width reflected the magnitude of Spearman’s ρ. Edge style indicated within-group correlation significance (solid = p < 0.05; dashed = non-significant). Edge color represented the direction of correlation change between groups, calculated using Fisher r-to-z transformation^40–42^ (red = strengthened correlation; blue = reduced correlation), and edge transparency represented the statistical support for that change, with more opaque edges indicating greater significance^43–46^.

In addition to validated gene interactions identified through Cytoscape interaction mapping, novel edges were incorporated when strong correlations were observed in the data. Specifically, new interactions were added when correlations exceeded |ρ| ≥ 0.9 and were statistically significant within either group, representing high-confidence associations not captured in curated interaction databases. Network topology was subsequently analyzed using the Cytoscape NetworkAnalyzer tool to quantify measures including node degree, connectivity, clustering coefficient, network density, and characteristic path length. Changes in node degree were used to identify genes that gained or lost connectivity following inclusion of newly identified interactions.

#### 2.7.5 Targeted protein analysis.

Protein targets were organized a *priori* into four biologically motivated categories to test focused hypotheses regarding AD–related pathology and circuit-relevant mechanisms in the amygdala. Proteins directly implicated in AD pathology were grouped as AD-related pathology and served as a targeted follow-up to validate immunohistochemical staining and transcriptional pathways identified in the gene expression analyses. A gliosis panel was selected to assess neuroinflammatory remodeling, including markers of both microglial and astrocytic activation. To evaluate potential alterations in neuronal signaling, proteins involved in excitatory and inhibitory neurotransmission were examined as molecular proxies of excitatory–inhibitory balance, given the well-established amygdala hyperactivity observed in PTSD.

Group differences between PTSD and age-matched control subjects were evaluated using the same statistical framework applied to the gene expression analyses. Prior to inferential testing, protein values were screened for statistical outliers in SPSS, and samples flagged as outliers were excluded from the corresponding protein-specific analyses. Assumptions of homogeneity of variance were evaluated using Levene’s test; when violated, group comparisons were performed using Welch’s t-test, and when not violated, standard independent-samples t-tests were used. Effect sizes (Cohen’s *d*) were calculated to quantify the magnitude of group differences. To account for multiple comparisons while preserving statistical power in targeted, hypothesis-driven analyses, false discovery rate (FDR) correction was applied using the same Benjamini–Hochberg procedure used for gene expression but implemented within each a *priori* biologically defined protein group (AD pathology, gliosis, excitation, inhibition). This group-wise approach balances control of Type I error with sensitivity to detect biologically meaningful effects within mechanistically coherent domains. Protein targets showing significant group differences were further evaluated using hierarchical linear regression, with diagnostic group (Control vs PTSD) entered in Model 1 and TBI status added in Model 2 to determine whether observed protein differences remained significant after accounting for TBI history.

#### 2.7.6 Postmortem Interval Quality Control Analyses.

To evaluate whether postmortem interval (PMI) contributed to variability in molecular and pathological measures, a series of quality control analyses were conducted prior to outlier removal. First, potential associations between PMI and quantitative immunohistochemical measures of pathology were assessed. Pearson correlation analyses were performed between PMI and amyloid burden (amyloid-positive pixel counts) as well as tau pathology (AT8-positive pixel counts), derived from image-based quantification of immunostained sections. Next, for gene expression data, principal component analysis (PCA) was performed across all measured genes using all available samples. A single principal component was extracted to capture the dominant shared variance across the gene dataset, and regression-based component scores (PC1) were calculated for each sample. These scores represent each sample’s position along the primary axis of global gene expression variability. Pearson correlation analysis was then used to assess the relationship between PC1 scores and PMI, providing a test of whether PMI explained meaningful variance in global gene expression. Finally, for protein analyses, total protein abundance values from all samples were analyzed prior to outlier removal to evaluate whether overall protein signal varied as a function of PMI. Pearson correlation analysis was performed to assess the relationship between total protein abundance and PMI. Together, these analyses were used to determine whether PMI explained variability in immunohistochemical pathology measures, global gene expression variance, or total protein abundance prior to downstream analyses.

## 3.0 Results

### 3.1 Cohort Demographics.

Postmortem amygdala tissue was obtained from 10 older adult donors, including 5 individuals with a lifetime diagnosis of PTSD and 5 age-matched controls without PTSD. All donors were male and identified as Caucasian, yielding a demographically homogeneous cohort with respect to sex and race (Table 1). Groups were closely matched for age. Mean age did not differ between PTSD and control donors, and Levene’s test confirmed homogeneity of variance (F = 0.118, p = 0.740). Under equal variances assumed, there was no evidence of an age difference between groups (t_(8)_ = − 0.196, p = 0.849).

Tissue quality metrics were comparable across diagnostic groups. RNA integrity number (RIN) values fell within the expected range for human postmortem brain tissue and did not differ systematically between PTSD and control cases (t_(8)_ = 0.772, p = 0.231; **Figure S1B**), supporting the suitability of these samples for targeted transcriptomic analyses (RT-qPCR). Postmortem interval (PMI) was numerically shorter in PTSD cases than in controls; however, PMI distributions overlapped substantially between groups and were within ranges commonly reported for postmortem molecular studies (Table 1). Although longer PMI can contribute to molecular degradation, such effects typically produce broad reductions in signal across proteins rather than selective alterations in specific pathways. In the present dataset, group differences were restricted to a subset of pathological and inflammatory markers, whereas many proteins were unchanged, arguing against generalized degradation in controls as the source of the observed effects. Together, these observations suggest that the differences detected between PTSD and control cases are unlikely to be explained by PMI-related degradation. Importantly, PMI did not predict the variability established by group differences (see below for details).

PTSD diagnoses were supported by clinical records and structured assessments, including the Clinician-Administered PTSD Scale (CAPS) where available. Control donors had no documented history of PTSD. No donors carried a diagnosis of a primary neurodegenerative disorder at the time of death. Reported history of traumatic brain injury (TBI) was infrequently and inconsistently documented across cases, with limited information regarding injury severity or timing. Accordingly, TBI variables were modeled as a controlling variable in linear regressions. Additional clinical and demographic characteristics are summarized in Table 1.

### 3.2 AD-Relevant Pathology in the Amygdala of Older Adults With PTSD.

**Figure 1:**
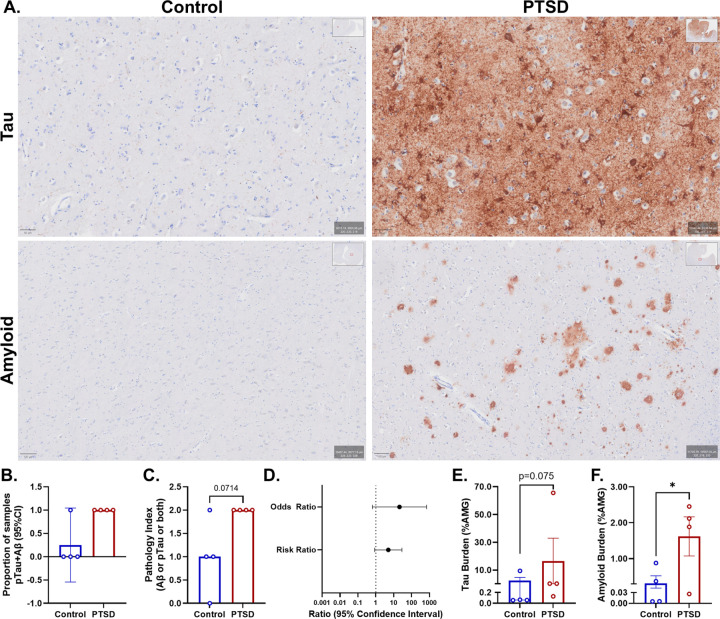
PTSD is associated with focal AD-like molecular pathology in the amygdala. **A:** We assessed AD-relevant pathology in the amygdala. **B-C:** Immunohistochemistry revealed colocalized pTau and amyloid in 4/4 PTSD cases versus 1/4 controls. **D:** 21-fold greater odds and 5-fold greater risk of AD-like pathology in PTSD. **E:** Although mean pTau burden did not differ, variance was significantly increased in PTSD (mean ±SEM). **F:** Amyloid burden was significantly elevated in PTSD relative to controls (mean ±SEM).

We next assessed AD–relevant pathology directly within the amygdala (Fig. 1A). Immunohistochemical analyses revealed that four of four evaluable PTSD cases exhibited colocalized AT8-positive phosphorylated tau and β-amyloid burden, whereas only one of four control cases demonstrated dual pathology (Fig. 1B).

To quantify this observation, we assigned a semiquantitative colocalization index (0 = no pathology; 1 = either AT8 or amyloid positivity; 2 = both pathologies present within the same sample; Fig. 1C). Median pathology index was higher in PTSD (median = 2.0, n = 4) compared with controls (median = 1.0, n = 4). This difference approached significance using an exact Mann–Whitney U test (U = 2, p = 0.0714), with a Hodges–Lehmann median difference estimate of 1.0 (97.14% CI: 0 to 2). Consistent with this ordinal analysis, contingency testing demonstrated enrichment of dual pathology in PTSD. Point estimates indicated that PTSD trended towards a 21-fold increase in the odds of exhibiting both tau and amyloid pathology (odds ratio = 21; 95% CI: 0.639–690) and a 5-fold increase in relative risk (risk ratio = 5; 95% CI:0.866–28.861; p = 0.071; Fig. 1D). Although confidence intervals were wide due to limited sample size, the magnitude and direction of effect were consistent across analytic approaches.

Continuous quantification of AT8 burden using percent DAB-positive pixels demonstrated marked heterogeneity in PTSD samples (Fig. 1E). Mean AT8 burden did not significantly differ between groups (t_(6)_ = 0.858, p = 0.2119), despite a large numerical separation in means (Control:2.409; PTSD: 16.57). Notably, variance was significantly greater in PTSD (F = 48.40, p < 0.01), indicating increased dispersion of tau burden within this group (Fig. 1E). Because AT8 burden violated assumptions of homogeneity of variance and exhibited consistent rank ordering between groups, a non-parametric Mann–Whitney U test was performed. Although this analysis did not reach statistical significance, PTSD cases showed higher rank values than controls (U = 3.00, p = 0.075), corresponding to a large effect size (rank-biserial correlation, r_rβ_=0.625). This pattern indicates a directional shift toward greater tau pathology in PTSD that was not fully captured by mean-based comparisons, and this was not explained by PMI (*ρ*=−0.371, p = 0.183). To determine whether tau differences extended beyond the amygdala, total AT8 burden including adjacent entorhinal cortex (EC) tissue was also analyzed. Although variance differed between groups (F = 5.99, p = 0.05), no group differences were observed using parametric testing (t_(6)_ = 0.78, p = 0.224). Consistent with this, a non-parametric Mann–Whitney U test revealed no differences in rank distribution between groups (U = 7.00, p = 0.443; r_rβ_=0.125), indicating a negligible effect size (**Figure S1C**). Notably, inclusion of EC signal shifted the distribution such that control cases exhibited comparable overall AT8 burden, suggesting that the previously observed elevation in PTSD is driven by amygdala-specific effects rather than global increases in tau pathology.

In contrast to tau, β-amyloid burden was significantly elevated in PTSD relative to controls in the full dataset (t_(6)_ = 2.243, p = 0.0330) (Fig. 1F). Mean amyloid burden expressed as the percentage of positive pixels in the amygdala was 0.3155 in controls and 1.623 in PTSD, corresponding to a mean difference of 1.307 ± 0.5827 and a moderate-to-large effect size (*d* = 1.59). Importantly, this effect was not explained by PMI (*ρ*=−0.228, p = 0.294). To determine whether TBI history influenced amyloid burden, hierarchical linear regression models were performed. In the initial model including only diagnostic group, PTSD status explained a substantial proportion of variance in amyloid staining (R^^2^^ = 0.456), with a positive association between PTSD and amyloid burden (β = 0.675, p = 0.033). Adding TBI status to the model did not significantly improve model fit (ΔR^^2^^ = 0.051, F-change = 0.52, p = 0.503), and TBI was not a significant predictor of amyloid staining (β = 0.378, p = 0.503). These findings indicate that the association between PTSD and amyloid burden was not explained by TBI history. To determine whether amyloid differences extended beyond the amygdala, total β-amyloid burden including adjacent EC tissue was also analyzed. Variance did not differ between groups (F = 1.09, p = 0.338), and therefore parametric testing was retained. No group differences were observed (t_(6)_ = 1.70, p = 0.071), although a directional trend toward higher amyloid burden in PTSD was present (**Figure S1D**). These findings indicate that, unlike tau, amyloid-related differences are not driven by variance-related effects and remain elevated when incorporating adjacent cortical regions.

Together, these findings indicate enrichment of focal AD-relevant pathology within the amygdala of older adults with PTSD, characterized by increased dual tau–amyloid positivity, elevated amyloid burden, and heterogeneous but potentially heightened tau remodeling.

### 3.3 Differential gene expression in the amygdala of older adults with PTSD.

**Figure 2:**
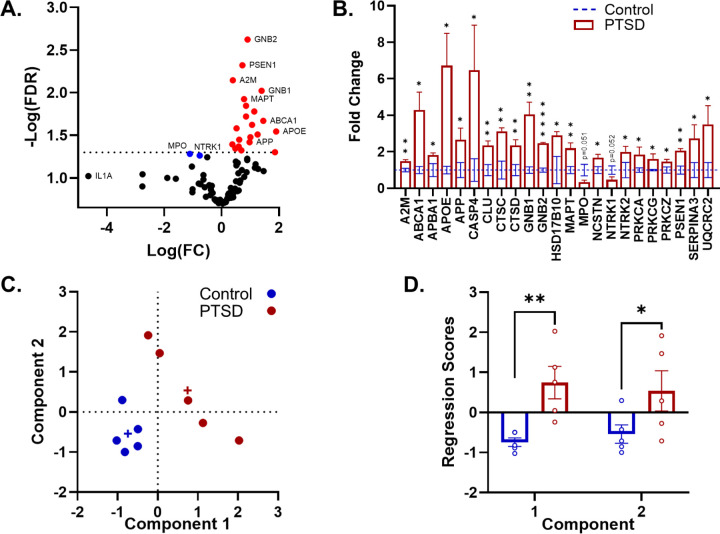
Coordinated AD-relevant transcriptional remodeling in PTSD amygdala. **A:** Differential expression analysis identified enrichment of AD-related genes in PTSD. **B:** Core amyloid/tau (APP, PSEN1, MAPT, NCSTN), lipid (APOE, ABCA1, CLU), and inflammatory/lysosomal genes were predominantly upregulated in PTSD. Data represent groups means ±SEM. **C:** The PTSD group segregated from controls across both gene components. **D:** Gene regression scores show significantly greater expression in PTSD relative to controls and consistent with our DEGs data in panel **B**.

To determine whether these histopathological differences were accompanied by coordinated molecular alterations within the amygdala, we next performed targeted transcriptional profiling of AD–relevant genes. Differential expression analysis identified a discrete subset of AD–related genes that differed between PTSD and age-matched control amygdala tissue at a nominal threshold of p < 0.05. Complete gene-level statistics, including fold change (FC), log fold change, t-values, degrees of freedom (df), p-values, rank-based false discovery rate–adjusted q-values, and Cohen’s d effect sizes, are reported in **Table S1**. Across all significant genes, effect sizes were predominantly moderate to large, and rank-based q-values remained well below the prespecified practical FDR threshold, indicating enrichment beyond random noise (Fig. 2AB; **Figure S1**). Importantly, a composite gene expression score representing global variability across genes was not significantly correlated with PMI (*ρ*=−0.286, p = 0.212).

Core components of amyloid and tau biology were significantly upregulated in the PTSD amygdala, including PSEN1, MAPT, APP, and the γ-secretase-associated gene NCSTN, indicating coordinated alterations in amyloidogenic processing pathways (see Table 4 for specific details and stats). Genes involved in lipid metabolism also showed robust upregulation, with large effect sizes observed for APOE, ABCA1, and CLU, consistent with enhanced lipid transport and amyloid-associated signaling. A parallel pattern emerged in inflammatory and lysosomal pathways. Multiple genes linked to immune activation and proteostasis (including A2M, CTSC, CTSD, SERPINA3, and CASP4) were significantly increased, supporting a shift toward heightened inflammatory and degradative activity within the amygdala. The strongest statistical effect was observed in G-protein signaling, with GNB2 showing the largest effect size in the dataset, alongside significant upregulation of GNB1, indicating prominent alterations in intracellular signaling cascades. Kinase-related signaling pathways were also affected, with increased expression of PRKCA, PRKCZ, and PRKCG, as well as the APP adaptor-related gene APBA1, suggesting downstream modulation of signaling pathways linked to synaptic and amyloid-related processes. Additional changes were observed in neurotrophic and metabolic pathways. NTRK2 was increased, and mitochondrial/metabolic genes HSD17B10 and UQCRC2 were also significantly elevated, indicating broader alterations in cellular energetics and neuronal support systems. Across all significant genes, differential expression was dominated by upregulation in PTSD. Only two genes, MPO and NTRK1, showed trend-level downregulation, and neither reached statistical significance.

### 3.4 PTSD-associated gene expression changes converge on coordinated AD-relevant molecular pathways in the amygdala.

To evaluate whether differentially expressed genes in the PTSD amygdala organize into coherent molecular modules, genes meeting the nominal significance threshold were fed into a principal component analysis (PCA). The PCA was performed on genes meeting the FDR threshold to reduce the dimensionality of the gene expression dataset and generate component scores representing coordinated transcriptional modules, allowing us to map group differences onto a gene-gene interaction network. After which, genes were then integrated into a gene–gene interaction network using Cytoscape. Two components were retained based on the scree criterion, explaining 58.2% and 12.7% of the total variance, respectively (71.0% cumulative variance explained). Gene communalities were generally high (> 0.5), indicating that the retained components captured the majority of shared variance across genes.

Consistent with our structured network in Fig. 2C, the first component was characterized by strong positive loadings encompassing lipid metabolism, inflammatory signaling, and proteostasis pathways (**Table S2**). The second component was dominated by a core amyloid–tau signaling network shown as a large increase in the correlational structure between APP and MAPT (Fig. 2C, **Table S2**). PTSD samples highly segregated from control samples (Fig. 2C; for component loadings and scores see **Table S2**). As such, multivariate analysis indicated strong separation between PTSD and control groups across the combined component structure (λ = 0.937, F_(2,7)_ = 51.90, p < 0.001). At the component level, group differences were significant for Component 1 (F_(1,8)_ = 12.75, p = 0.0035, η^^2^^=0.614; Fig. 2D) and Component 2 (F_(1,8)_ = 3.81, p = 0.044, η^2^=0.322; Fig. 2D).

**Figure 3:**
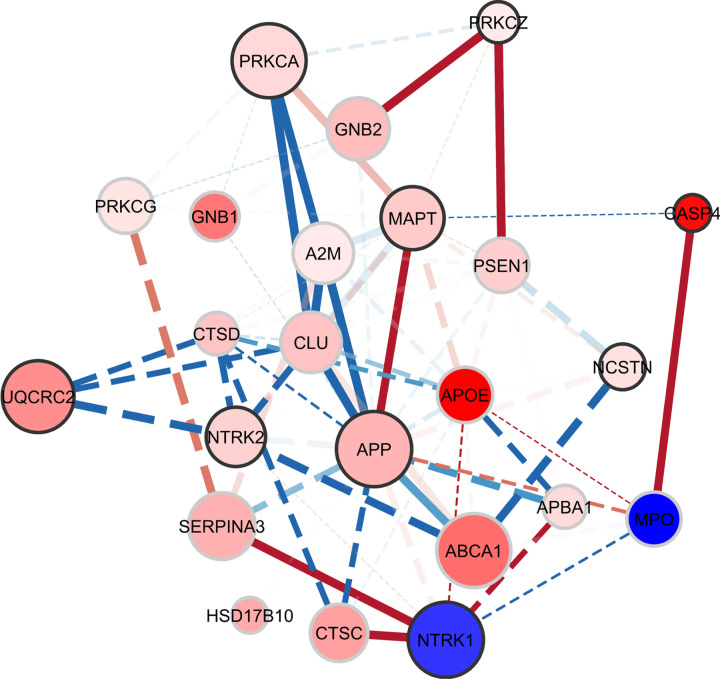
AD-related gene network remodeling in PTSD amygdala. Gene network analysis revealed a compact, APP-centered architecture with strong connectivity to PSEN1, MAPT, and APOE, consistent with canonical AD pathway organization. Nodes are scaled by loading score and color-coded by fold-change in PTSD relative to controls (red=increase, blue=decrease). Edges represent gene-gene correlations, with thickness scaled to |*ρ*| (thickest near 1.0, thinnest near 0.0); line type indicating p-value (solid<0.1, dashed≥0.1), color denoting Z-score changes in correlation strength between PTSD and Control (red=positive change, blue=negative change), and transparency reflecting the p-value of the Z-score change (more opaque=more significant).

Network topology statistics indicated a compact and well-connected structure. The resulting network consisted of 23 nodes connected by 77 edges, with an average of 6.696 neighbors per node, indicating substantial connectivity among differentially expressed genes. The network exhibited a diameter of 3 and a radius of 2, reflecting a compact topology in which most genes were separated by only one to two interaction steps. The characteristic path length was 1.818, consistent with efficient communication across the network. The network showed a clustering coefficient of 0.529 and a density of 0.304, indicating moderate local clustering with greater overall connectivity than expected for a sparsely connected system. Measures of network heterogeneity (0.553) and centralization (0.513) suggested a partially hub-driven architecture in which a subset of genes contributed disproportionately to network connectivity. The network contained a single connected component, indicating that all genes were integrated within a unified interaction structure. Complete node-level topology metrics and edge-level statistics describing correlation structure and interaction changes are reported in **Tables S2 and S3**, respectively. Network visualization is shown in Fig. 3.

Analysis of node-level topology revealed a hub-centered network architecture dominated by amyloid-associated genes. APP emerged as the primary network hub, exhibiting the highest connectivity (degree = 17) and the greatest betweenness centrality (0.307), indicating a central role in coordinating interactions across the network. APP also showed the highest closeness centrality (0.815), consistent with its position as the most globally integrated node. APOE and MAPT formed a second tier of highly connected nodes, with degrees of 13 and 12, respectively. MAPT displayed relatively high betweenness centrality (0.152), suggesting a strong brokerage role linking multiple network regions, whereas APOE exhibited high connectivity with comparatively lower brokerage, consistent with a major connector within lipid and amyloid-associated pathways. CLU represented the next most connected node (degree = 11) and showed moderate betweenness centrality (0.073), positioning it as an additional coordinator within the core interaction structure. Several additional AD–associated genes occupied intermediate connectivity positions, including CTSD and PSEN1 (degree = 9 each). Both genes exhibited moderate betweenness centrality, indicating participation in routing interactions between major network modules.

A third tier of nodes showed moderate connectivity, including A2M (degree = 8) and several signaling or neurotrophic genes such as NTRK2, PRKCA, and NTRK1 (degree = 7 each). These nodes displayed lower betweenness centrality values but remained integrated within the broader interaction structure, suggesting roles in linking signaling pathways to the amyloid-centered network core. Overall, node topology indicated that the PTSD-associated amygdala gene network is strongly organized around an APP-centered hub, with APOE, MAPT, and CLU forming secondary hubs that coordinate interactions across lipid transport, proteostasis, and intracellular signaling pathways.

Edge statistics revealed extensive correlation structure among differentially expressed genes in the PTSD amygdala. The expanded network contained 77 edges, reflecting both validated STRING interactions and additional high-confidence correlations identified in the dataset. Edge width represented the magnitude of Spearman correlations, with thicker edges corresponding to stronger gene–gene associations. Several interactions showed very strong correlations (|ρ| ≥ 0.8), indicating tightly coupled expression relationships among key genes. The strongest correlations were concentrated around APP, CLU, A2M, PSEN1, and GNB2, reinforcing the central position of amyloid- and proteostasis-related pathways in the network. In particular, multiple APP-centered interactions ranked among the highest correlations, supporting its role as the dominant hub coordinating interactions across the network.

Correlation changes between groups further revealed substantial network reorganization. Fisher r-to-z analysis identified several edges exhibiting large changes in correlation strength, with the most pronounced shifts involving interactions linking APP, CLU, A2M, APOE, CTSD, and MPO. These changes indicate that PTSD-associated transcriptional alterations modify the coordination between genes involved in amyloid processing, lipid transport, inflammatory signaling, and lysosomal proteostasis. Edges involving APP and CLU showed some of the largest changes in correlation strength, consistent with their roles as major structural hubs in the network. Similarly, interactions involving A2M and CTSD demonstrated notable correlation shifts, suggesting altered coupling within inflammatory and proteostatic pathways. Changes in edges associated with APOE further linked lipid transport processes to the core amyloid-centered network architecture.

The inclusion of strong data-driven correlations substantially increased network connectivity relative to the validated interaction network alone, expanding the total number of edges from 55 to 77 and increasing the average number of neighbors per node. Despite this increase in connectivity, the network remained compact, with most genes connected through a small number of intermediate interactions. Together, these edge-level statistics indicate that PTSD-associated transcriptional changes in the amygdala not only alter gene expression levels but also reshape the correlation structure linking genes across amyloid, lipid, inflammatory, and signaling pathways, reinforcing a highly interconnected molecular system centered on APP and its associated regulatory networks (Fig. 3).

To determine whether the observed transcriptional differences between PTSD and control groups were influenced by TBI history, regression scores from the PCA components were entered into hierarchical linear regression models. In each analysis, diagnostic group was entered in the first model and TBI status was added in the second model. For Component 1, which captured the inflammatory and lipid-associated transcriptional module, diagnostic group explained a substantial proportion of variance in component scores (R^^2^^=0.614, F_(1,8)_ = 12.75). The group effect was significant (p = 0.0035), with PTSD cases exhibiting higher component scores than controls (β = 0.784). Adding TBI status to the model did not significantly improve model fit (ΔR^^2^^=0.007, F-change = 0.13, p = 0.364), and TBI was not a significant predictor of Component 1 scores (β=−0.141, p = 0.364). These results indicate that the group difference captured by Component 1 was not explained by TBI history. For Component 2, representing the amyloid–tau signaling module, the initial model showed a moderate association with diagnostic group (R^^2^^=0.322, F_(1,8)_ = 3.81; p = 0.044). However, adding TBI status did not significantly improve model fit (ΔR^^2^^=0.003, F-change = 0.036, p = 0.428), and TBI was not a significant predictor of Component 2 scores (β = 0.098, p = 0.428). Together, these analyses indicate that the transcriptional patterns captured by the PCA components distinguish PTSD and control groups and that these differences are not attributable to TBI history.

### 3.5 PTSD is associated with proteins directly implicated in Alzheimer’s disease pathology.

**Figure 4:**
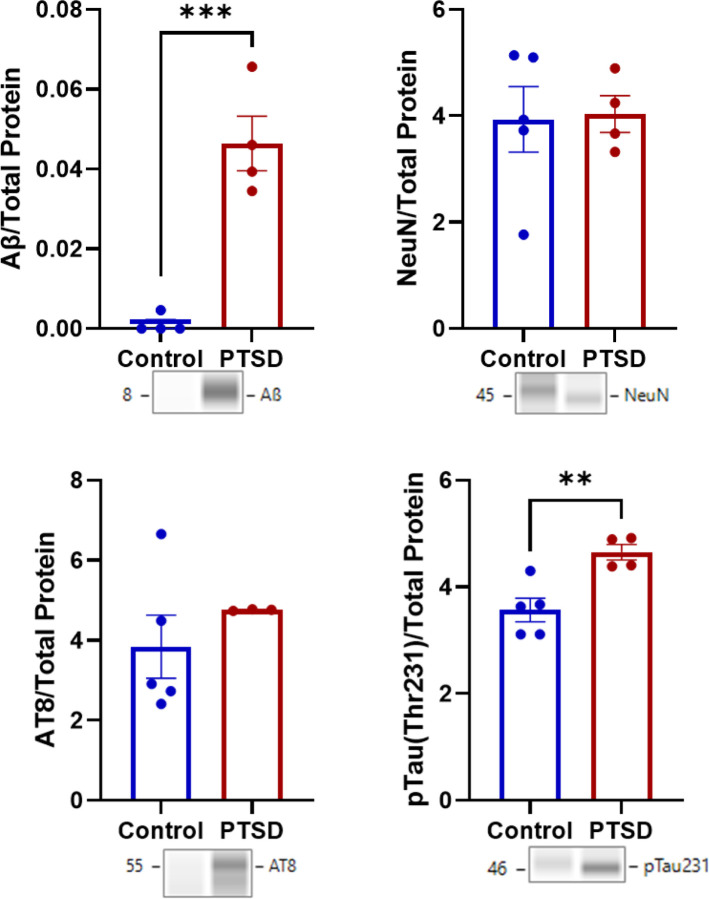
AD-related protein remodeling in PTSD amygdala. PTSD was associated with elevated pTau231 and increased amyloid-β, whereas total tau (AT8) was numerically increased, indicating site-specific tau phosphorylation rather than global accumulation. NeuN levels did not differ between groups, demonstrating that AD-relevant molecular alterations occurred in the absence of overt neuronal loss. Data represents group means ±SEM.

Among AD pathology-related proteins, pTau231 was robustly elevated in PTSD (t_(7)_ = 3.878, p = 0.003; *d* = 2.601; Fig. 4, **Table S4**). Additionally, Aβ was significantly higher (t_(6)_ = 6.519, p < 0.001; *d* = 4.610). However, AT8 was not significantly different (t_(6)_ = 0.872, p = 0.155), suggesting no differences in total tau protein despite higher phosphorylation at specific AD-relevant sites in the PTSD group (pTau231). The neuronal marker NeuN did not differ between groups (t_(7)_ = 0.131, p = 0.450), suggesting no detectable group difference in neuronal marker abundance at the protein level in this cohort.

To determine whether observed protein differences between groups were influenced by traumatic brain injury (TBI) history, proteins showing group differences were evaluated using two-model linear regressions. Model 1 included group (Control vs PTSD), and Model 2 added TBI history as a covariate. For amyloid-β, group significantly predicted amyloid burden in the amygdala (Model 1: F_(1,6)_ = 42.50, R^2^ = 0.876, p < 0.001). PTSD cases exhibited higher amyloid levels than controls (β = 0.936). Adding TBI did not significantly improve the model (ΔR^2^ = 0.022, F-change_(1,5)_ = 1.11, p = 0.170), and TBI was not a significant predictor (p = 0.170). The group effect remained significant after accounting for TBI (β = 1.137, p = 0.0025). A similar pattern was observed for pTau-Thr231. Group significantly predicted phosphorylated tau levels (Model 1: F_(1,7)_ = 15.04, R^2^ = 0.682, p = 0.003), with PTSD associated with higher pTau231 expression (β = 0.826). Inclusion of TBI did not significantly improve model fit (ΔR^2^ = 0.009, p = 0.348), and TBI was not significant (p = 0.348). The group effect remained significant after adjustment for TBI (β = 0.951, p = 0.023).

To determine whether group differences in pathology-related proteins could reflect variation in total protein loading, total protein values corresponding to these assays were compared between PTSD and control groups. No significant group differences were detected for loading controls associated with Aβ, AT8, pTau(Thr231), or NeuN (ps = 0.121–0.327), indicating that the observed pathology-related protein differences were unlikely to be explained by variation in total protein loading. Representative electropherograms for total protein per group are shown in **Figure S2–4**.

### 3.6 Evidence for gliosis in the amygdala of older adults with PTSD.

**Figure 5:**
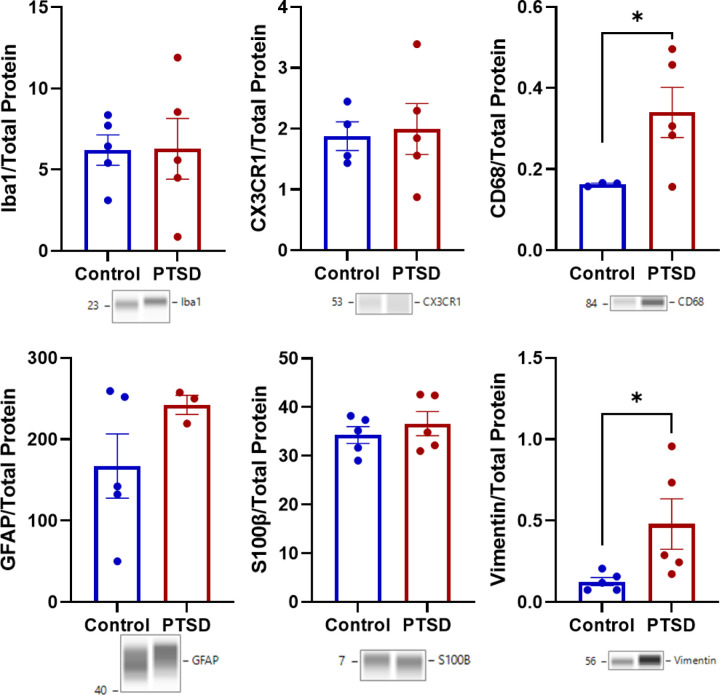
Selective glial remodeling in PTSD amygdala. PTSD was associated with elevated CD68 and vimentin, indicating increased microglial activation and astrocytic remodeling (mean ±SEM). In contrast, GFAP and S100β were unchanged, suggesting selective inflammatory remodeling rather than diffuse gliosis.

Within microglia-related protein, CD68 was higher in PTSD (t_(6)_ = 2.513, p = 0.023; *d* = 1.572; Fig. 5, **Table S4**). Other microglial and immune markers were not different, including Iba1 (t_(8)_ = 0.035, p = 0.973) and CX3CR1 (t_(7)_ = 0.225, p = 0.414). Astrocytic markers were also unchanged, including GFAP (t_(6)_ = 1.409, p = 0.104; *d* = 1.029) and S100β (t_(8)_ = 0.765, p = 0.466). Vimentin, however, was greater in PTSD (t_(8)_ = 2.252, p = 0.042; *d* = 1.424).

For Vimentin, group showed a trend toward higher expression in PTSD in the initial model (Model 1: F_(1,8)_ = 5.07, R^2^ = 0.388, p = 0.027). However, after inclusion of TBI, the group effect was no longer significant (p = 0.313), and TBI itself was not associated with Vimentin expression (p = 0.164). For CD68, the initial model showed a group effect (Model 1: F_(1,6)_ = 4.63, R^2^ = 0.436, p = 0.038). After accounting for TBI, the group effect was no longer significant (p = 0.067), and TBI was not associated with CD68 levels (p = 0.257).

Total protein values corresponding to glial markers also did not differ between groups. Loading controls associated with Iba1, CX3CR1, CD68, GFAP/S100β, and Vimentin were not significantly different between PTSD and control cases (ps = 0.155–0.355), indicating that group differences in glial protein abundance were not attributable to systematic variation in total protein loading.

### 3.7 Neuronal markers of excitation are more abundant in the amygdala of older adults with PTSD, whereas no group differences in markers of inhibition.

**Figure 6:**
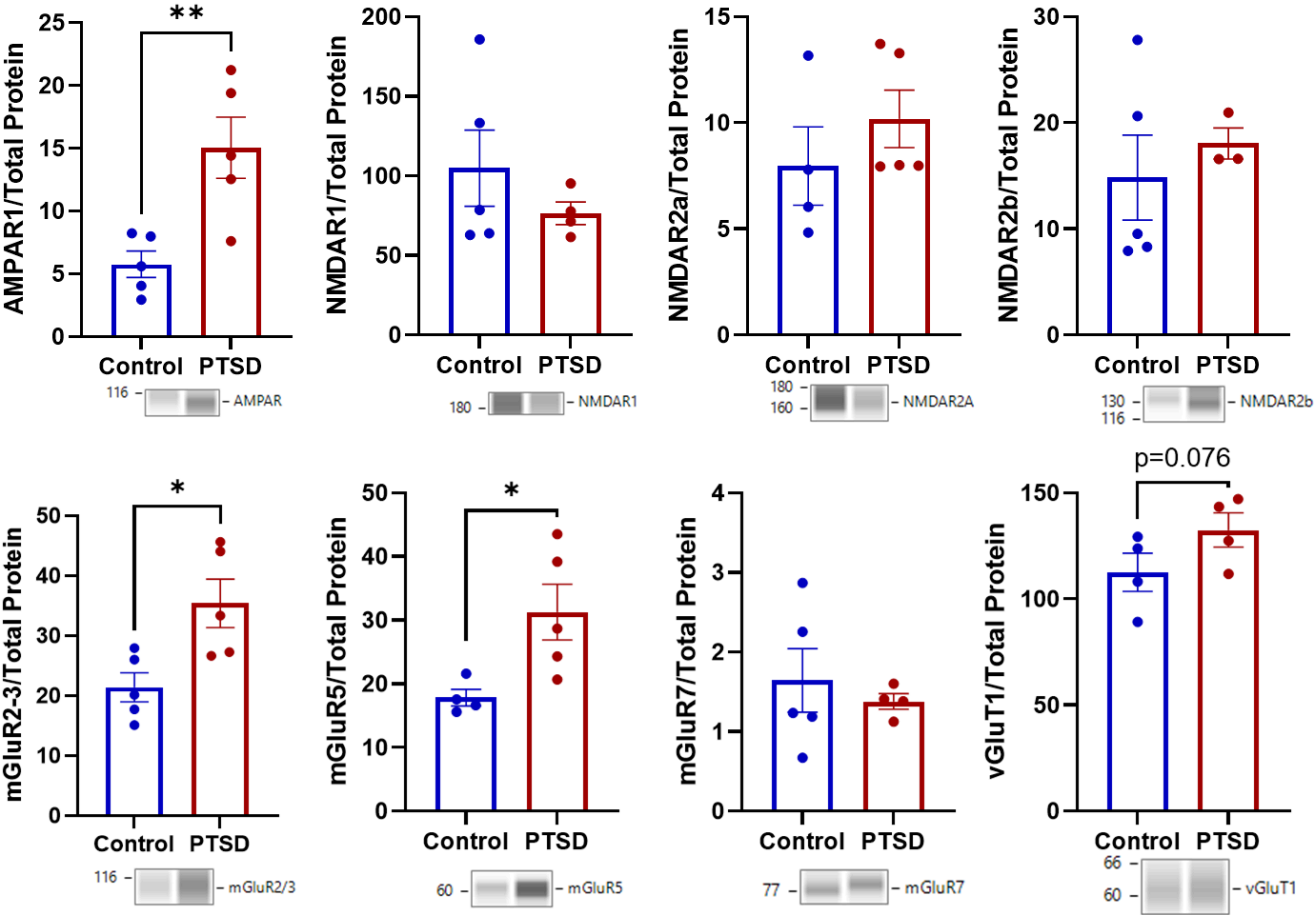
Elevated markers of excitatory synaptic signaling in the amygdala of older combat Veterans with PTSD. Quantification of synaptic proteins revealed selective increases in excitatory signaling components in the PTSD group relative to age-matched controls. AMPAR1 protein abundance was elevated in PTSD, and metabotropic glutamate receptors mGluR2/3 and mGluR5 were also increased. The presynaptic glutamate transporter vGluT1 showed a trend toward higher expression in PTSD. In contrast, other excitatory synaptic targets (including NMDAR1, NMDAR2a, NMDAR2b, and mGluR7) did not differ between groups. Data represent (mean ±SEM) group comparisons of protein abundance in postmortem amygdala tissue from older combat Veterans with PTSD and age-matched controls. Full statistical results are reported in Table S4.

Markers of excitatory signaling showed the clearest synaptic differences. AMPAR1 was significantly higher in PTSD (t_(8)_ = 3.492, p = 0.008; *d* = 2.209; Fig. 6, **Table S4**), and mGluR2/3 (t_(8)_ = 2.967, p = 0.009; *d* = 1.876) and mGluR5 (t_(8)_ = 2.644, p = 0.017; *d* = 1.774) were also higher. There was also a trend toward greater vGluT1 protein in PTSD (t_(6)_ = 1.641, p = 0.076 *d* = 1.160). Other excitatory targets did not differ, including NMDAR1, NMDAR2a, NMDAR2b, mGluR7, and (ts(6−7)=−1.018–0.990, ps = 0.155–0.276).

**Figure 7:**
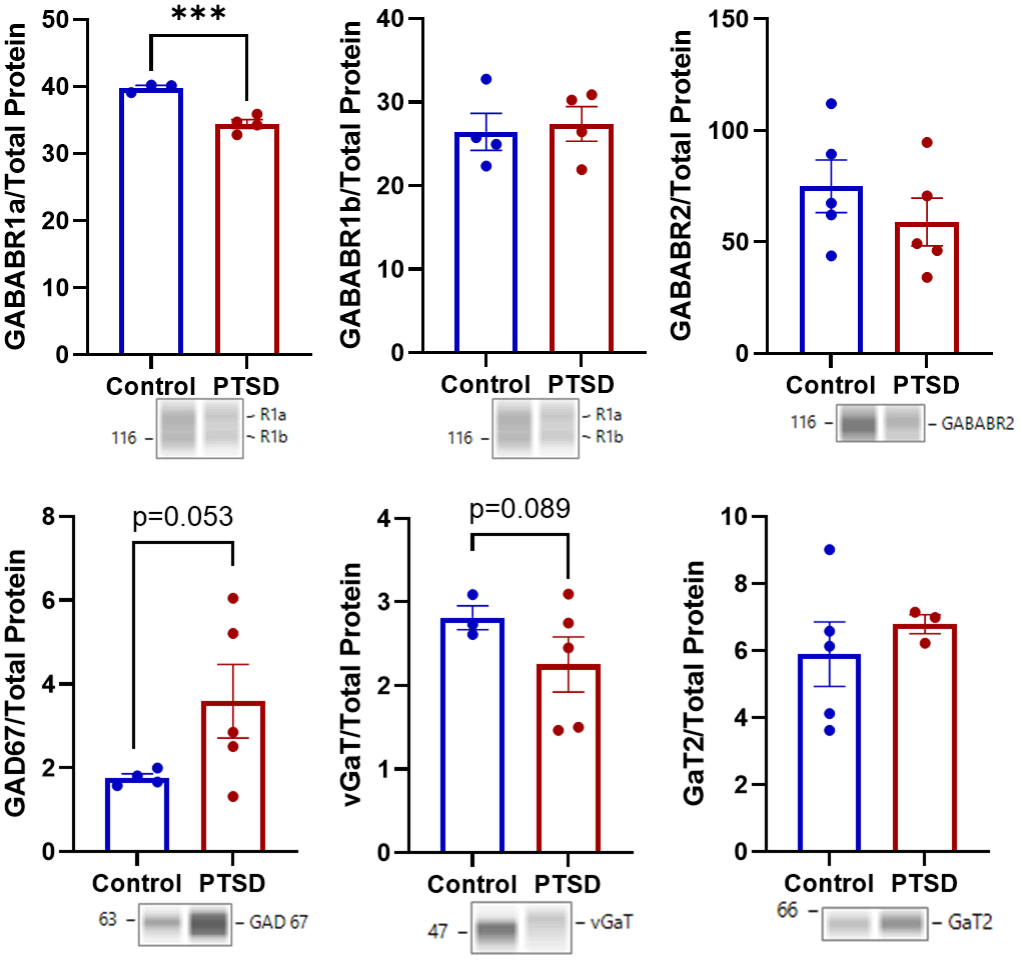
Selective alterations in inhibitory synaptic markers in the amygdala of older combat Veterans with PTSD. Quantification of inhibitory synaptic proteins revealed selective differences between PTSD and control groups. The R1a isoform of the GABAB receptor subunit GABABR1 was reduced in PTSD, while the vesicular GABA transporter (vGAT) showed a trend toward lower abundance. In contrast, the GABA synthesis enzyme GAD67 showed a trend toward higher expression in PTSD. Other inhibitory synaptic proteins (including GABABR1b, GABABR2, and GAT2) did not differ between groups. Data represent group comparisons of protein abundance in postmortem amygdala tissue from older combat Veterans with PTSD and age-matched controls (mean ±SEM). Full statistical results are reported in Table S4.

Interestingly, the R1a isoform of GABABR1 was significantly lower in PTSD (t_(5)_=−6.573, p < 0.001; d=−5.020; Fig. 7, **Table S4**), and vGaT trended lower (t_(6)_=−1.236, p = 0.089; d=−0.902), whereas GAD67 was trending higher (t_(7)_ = 1.821, p = 0.053; d = 1.222). Other inhibitory proteins were not significantly different between groups. This included GABABR1b, GABABR2, and GaT2 (ts(6−8)=−1.003–0.686, ps = 0.173–0.384).

For AMPAR1, group significantly predicted protein levels (Model 1: F_(1,8)_ = 12.20, R^2^ = 0.604, p = 0.004), with PTSD cases showing higher AMPAR1 expression (β = 0.777). After including TBI, the group effect was attenuated and no longer significant (p = 0.088), and TBI was not associated with AMPAR1 expression (p = 0.277). Similarly, mGluR2/3 expression was significantly predicted by group in the initial model (Model 1: F_(1,8)_ = 8.80, R^2^ = 0.524, p = 0.009), with higher levels observed in PTSD (β = 0.724). However, after controlling for TBI, the group effect was no longer significant (p = 0.215), and TBI itself was not significant (p = 0.128). For mGluR5, group significantly predicted expression in the initial model (Model 1: F_(1,7)_ = 6.99, R^2^ = 0.500, p = 0.0165). After inclusion of TBI, both group (p = 0.0065) and TBI (p = 0.048) showed associations with expression, suggesting partial shared variance between group status and TBI history in explaining mGluR5 levels. For GABABR1a, group strongly predicted protein expression (Model 1: F_(1,5)_ = 43.21, R^2^ = 0.896, p < 0.001), with PTSD cases showing lower expression than controls (β=−0.947). Inclusion of TBI did not alter model fit (ΔR^2^ ≈ 0, p = 0.493), and TBI was not associated with expression (p = 0.493). The group effect remained significant after adjustment (β=−0.942, p = 0.0125). In contrast, vGluT1, GAD67, and vGAT did not show significant group effects in the initial models (p = 0.076, p = 0.056, and p = 0.132, respectively), and inclusion of TBI did not significantly improve model fit or explain additional variance in expression. Collectively, these analyses indicate that amyloid-β, pTau-Thr231, GABABR1a, and mGluR5 group differences remain robust after accounting for TBI, whereas differences in glial markers and several excitatory synaptic proteins were attenuated when TBI was included as a covariate.

For proteins associated with synaptic excitation and inhibition, most loading controls likewise did not differ between groups (ps = 0.108–0.494). While total protein loaded for mGluR2/3 trended toward a difference (p = 0.063), this was driven by n = 1 sample and removing the sample did not change interpretations of group differences, thus the difference (PTSD>Control) was stable (**Figure S3** shows typical electropherograms for mGluR2/3). The only difference was NMDAR1, for which total protein values differed between groups (t_(8)_ = − 3.71, p = 0.003). Thus, aside from this isolated case, the observed synaptic protein differences were unlikely to be explained by differences in total protein loading. Importantly, a composite total protein score representing global variability across all protein targets was not significantly correlated with PMI (*ρ*=−0.036, p = 0.920), suggesting that observed group differences in protein measures were unlikely to be explained by PMI.

## 4.0 Discussion

The present study provides convergent evidence that PTSD in older adults is associated with Alzheimer’s disease (AD)-relevant remodeling in the human amygdala. Across histopathology, targeted transcriptional profiling, network analysis, and protein quantification, older donors with PTSD showed greater amyloid burden, enrichment of combined tau–amyloid positivity, coordinated upregulation of AD-related genes, selective gliosis-associated changes, and synaptic protein alterations consistent with a shift toward excitatory dominance and reduced inhibitory restraint. These findings are notable because PTSD has repeatedly been linked to accelerated cognitive aging and elevated risk for dementia in Veteran and non-Veteran cohorts^4–6^. Rather than reflecting only broad psychiatric burden, our data suggest that late-life PTSD is associated with a biologically coherent amygdala signature that overlaps with molecular pathways implicated in AD-related vulnerability.

This interpretation is consistent with the broader clinical literature. Older Veterans show measurable cognitive decline relative to the general population^47^, and this decline appears steeper in those with PTSD^7^. PTSD in aging populations has been associated with impairments across multiple cognitive domains, including episodic memory, attention, and executive function^8^. Recurrence or re-emergence of PTSD symptoms has also been documented in late life even after long periods of remission^48^. Importantly, PTSD-related executive dysfunction in cognitively normative older adults can approach the magnitude observed in mild cognitive impairment^9^. In parallel, neuroimaging studies have reported tau-related abnormalities in Veterans with chronic PTSD, including increased tau accumulation that correlates with amyloid burden^10^. Our findings extend those observations by demonstrating that the amygdala itself exhibits a postmortem molecular and pathological profile consistent with increased AD-relevant vulnerability in older adults with PTSD.

One of the clearest findings in the present study was the enrichment of AD-relevant pathology within the amygdala of PTSD cases. PTSD donors showed greater β-amyloid burden and a higher frequency of dual tau–amyloid positivity. Although continuous AT8 burden did not significantly differ between groups, PTSD cases showed marked heterogeneity in AT8-positive area together with a robust increase in pTau231 at the protein level. This pattern suggests that tau-related remodeling in PTSD may be more sensitively captured by phosphorylation at specific AD-relevant epitopes rather than by total tau burden assessed using a single marker across a small cohort. Notably, our findings are consistent with prior postmortem work showing that military service is associated with a greater incidence of neuritic amyloid plaques^49^. However, that study did not include PTSD as a group variable and did not assess region-specific pathology, limiting interpretation with respect to trauma-related mechanisms and circuit-level vulnerability.

Our findings provide important context for prior PET imaging studies examining AD-related pathology in Veterans with PTSD, which have yielded mixed results. Some studies report no significant increase in global amyloid burden in PTSD alone^12,13^, although one of these demonstrated elevated amyloid signal specifically in individuals with combined PTSD and TBI, suggesting that additional biological stressors may unmask detectable pathology^12^. The Marcolini study, which reported null findings, was further limited by relatively small sample sizes^13^. In contrast, other PET studies have identified increased tau and amyloid signal in specific brain regions of Veterans with PTSD, with effects that track with disease-relevant pathology^10,11,50^. Taken together, these discrepancies may reflect methodological differences rather than true absence of pathology. PET imaging relies on spatially averaged signal across relatively large regions of interest and has limited resolution for detecting subtle or regionally restricted changes, particularly within small structures such as the amygdala. As a result, localized or early-stage molecular alterations may fall below the detection threshold of in vivo imaging approaches. In contrast, the present study employs regionally precise, postmortem molecular and histopathological analyses, enabling detection of pathology at a level not accessible through PET imaging. Indeed, the amygdala may be particularly susceptible to such remodeling because it plays a central role in emotional salience and threat processing, functions that are consistently dysregulated in PTSD^14–16^. Basolateral amygdala circuits are critical for fear learning, extinction memory, and adaptive threat regulation, and dysfunction within these circuits has been strongly implicated in PTSD-related psychopathology^14–17^. Within this context, the presence of AD-relevant pathological signatures in the amygdala may reflect the cumulative biological consequences of chronic trauma-related circuit dysregulation.

The transcriptional findings strongly reinforce this interpretation. Differentially expressed genes in the PTSD amygdala converged on pathways classically implicated in AD biology, including amyloid processing, tau signaling, lipid metabolism, inflammatory activation, lysosomal function, and proteostasis. Upregulation of genes such as APP, MAPT, PSEN1, NCSTN, APOE, ABCA1, CLU, CTSD, CTSC, A2M, and CASP4 suggests activation of a coordinated molecular program rather than isolated gene-level effects. These pathways collectively represent central biological domains implicated in AD pathogenesis, which is increasingly understood as a systems-level disorder involving interactions among amyloid metabolism, lipid homeostasis, inflammatory signaling, and protein degradation pathways^19–24^. The predominance of transcriptional upregulation across these genes further suggests that PTSD is associated with a stress-responsive or activated molecular state in the aging amygdala that may facilitate pathological remodeling.

Network analysis further demonstrated that PTSD was associated with substantial reorganization of the relationships among AD-related genes. APP emerged as the dominant network hub, with APOE, MAPT, and CLU forming a second tier of highly connected nodes. At the component level, the transcriptional architecture segregated into modules associated with inflammatory–lipid signaling and amyloid–tau interactions. This hub-centered organization suggests that PTSD may reshape the regulatory architecture of molecular pathways within the amygdala in a way that amplifies interactions among AD-relevant biological systems. APP-centered network organization is particularly notable because APP sits at the intersection of amyloid processing, synaptic regulation, and stress-responsive cellular signaling pathways. These findings therefore support the idea that PTSD is associated not only with differential gene expression but also with altered coordination among molecular pathways relevant to neurodegeneration.

Inflammatory processes likely contribute to this remodeling. Chronic inflammation has long been implicated in both neuropsychiatric disorders and neurodegenerative diseases^19–23^. PTSD has been associated with elevated inflammatory signaling and immune dysregulation34, and inflammation itself can directly influence amygdala activity and threat processing^21,22^. More broadly, aging is accompanied by a progressive shift toward a proinflammatory systemic state, often termed “inflammaging,” which has been proposed to contribute to cognitive decline and increased neurodegenerative vulnerability^23–26^. Elevated inflammatory cytokines, including interleukin-6, increase with age and have been associated with functional decline and adverse health outcomes in older populations^27–33^. In the present study, PTSD donors exhibited increased CD68 and vimentin protein expression alongside transcriptional upregulation of several lysosomal and inflammatory genes. At the same time, other canonical glial markers such as Iba1, CX3CR1, GFAP, and S100β were unchanged. This pattern argues against uniform glial expansion and instead suggests selective inflammatory remodeling, potentially reflecting altered microglial phagolysosomal activity or reactive structural remodeling rather than generalized gliosis.

Alterations in synaptic signaling provide an additional mechanistic layer linking PTSD to amygdala dysfunction. PTSD cases showed higher expression of multiple excitatory synaptic markers, including AMPAR1, mGluR2/3, and mGluR5, together with a pronounced reduction in the inhibitory receptor subunit GABABR1a. Although not all excitatory and inhibitory markers differed between groups, the overall pattern is consistent with a shift toward increased excitatory signaling and reduced capacity for inhibitory regulation. Such a shift aligns with longstanding models of PTSD that emphasize amygdala hyperactivity and exaggerated threat responsivity^14–16^. Importantly, similar forms of circuit instability have also been observed in preclinical models of early AD-related pathology, where neuroinflammation and synaptic dysfunction contribute to network hyperexcitability and behavioral impairment^34,35^, including our own work^36,37^. Within this framework, the reduction in GABABR1a is particularly noteworthy, as GABAB signaling plays an important role in regulating synaptic excitability and maintaining inhibitory tone. Indeed, if these reductions preferentially affect presynaptic glutamatergic terminals, GABAB-mediated control of glutamate release would be diminished, further biasing the system toward excessive excitatory signaling. A state characterized by enhanced excitatory signaling combined with reduced inhibitory restraint could therefore promote persistent threat salience and maladaptive plasticity in the aging amygdala.

Taken together, the pathology, transcriptional, inflammatory, and synaptic findings support an integrative model in which chronic PTSD contributes to a biological environment within the amygdala that is permissive for AD-relevant remodeling. In this model, repeated trauma-related activation and chronic stress physiology interact with aging-associated inflammatory and metabolic changes to promote amyloidogenic signaling, tau phosphorylation, proteostatic stress, and altered synaptic regulation. Aging itself is accompanied by progressive inflammatory and metabolic shifts that increase susceptibility to neurodegenerative processes^23–26^. PTSD may therefore act as a chronic systems-level stress state that biases vulnerable neural circuits toward pathological aging trajectories. The amygdala, given its central role in emotional salience and stress responsivity, may represent one such vulnerable node.

An important strength of this study is that the primary findings were not readily explained by common postmortem confounds. Postmortem interval did not account for pathology burden, global gene-expression variance, or total protein variability. Similarly, traumatic brain injury (TBI) history did not explain the most robust findings, including amyloid burden, pTau231 levels, the principal transcriptional components, or GABABR1a expression. This distinction is important because prior imaging studies have suggested that both PTSD and TBI can contribute to tau-related abnormalities in Veterans^10^. While our dataset cannot fully disentangle the long-term contributions of PTSD and TBI, the present findings indicate that the core molecular signal observed in the amygdala cannot be attributed solely to TBI history. Nonetheless, some glial and excitatory protein effects were attenuated after accounting for TBI, suggesting that certain inflammatory or synaptic markers may reflect overlapping influences of trauma and injury.

### 4.1 Limitations.

Several limitations should be acknowledged. The cohort size was necessarily small, all donors were older Caucasian men, and the study design was cross-sectional and postmortem. These features limit statistical power and generalizability and preclude causal inference. Some pathology analyses were based on only four evaluable cases per group, and confidence intervals around categorical pathology estimates were correspondingly wide. Formal Braak staging and ABC scoring were not available for these cases, and detailed lifetime clinical histories (including medication exposure, vascular disease, and precise TBI severity) could not be comprehensively modeled. In addition, the amygdala was analyzed as a single region, preventing resolution of potential subnucleus-specific effects. Despite these limitations, the convergence of findings across histopathology, transcriptional profiling, network analysis, and protein quantification strengthens the interpretation that PTSD is associated with coordinated molecular remodeling in the aging amygdala.

### 4.2 Conclusion.

The translational implications of these findings are notable. Epidemiological studies have consistently demonstrated elevated risk of dementia in individuals with PTSD^4–6^, yet the regional biological mechanisms underlying this association remain poorly understood. The present results suggest that the amygdala may represent a key anatomical site where trauma-related circuit dysregulation intersects with age-related neurodegenerative pathways. Future studies in larger and more diverse cohorts will be required to determine whether these molecular signatures track symptom severity, cognitive decline, or progression to clinical dementia. Understanding how trauma-related psychiatric illness interacts with biological aging processes may ultimately help identify therapeutic targets aimed at stabilizing vulnerable neural circuits before irreversible neurodegeneration occurs.

In conclusion, older adults with PTSD exhibited convergent evidence of AD-relevant pathological and molecular remodeling in the amygdala. Increased amyloid burden, elevated pTau231, APP-centered transcriptional network reorganization, selective inflammatory remodeling, and synaptic signatures of excitatory bias collectively support a model in which PTSD is associated with a late-life amygdala state that overlaps with mechanisms of pathological brain aging. These findings strengthen the view that trauma-related psychiatric illness may contribute to neurodegenerative vulnerability through regionally specific molecular remodeling within circuits that govern emotional salience and threat processing.

## Supplementary Material

This is a list of supplementary files associated with this preprint. Click to download.

- TableS1.xlsx
- TableS2.xlsx
- TableS3.xlsx
- TableS4.xlsx
- SFiguresHumanBLA2026Submit.pdf

## Figures and Tables

**Table 1:** Cohort Demographics.

ID	Age	Sex	Race	Primary dx	PMI	Laterality Frozen	Laterality Fixed	Branch	Combat exposure	History TBI	Neuro dx	Psych dx
8708	71	M	CAUC	Control	47.5	L5	R7	Unknown	Yes	No	No	No
8099	74	M	CAUC	Control	45.88	L8	R7	Army	Yes	Yes	No	history of MDE
8111	70	M	CAUC	Control	43.67	R4	L7	Unknown	Unknown	No	No	Alcohol Use Disorder
9974	72	M	CAUC	Control	35.42	L6	R8	Unknown	Unknown	No	No	No
7497	73	M	CAUC	Control	26.7	L6	R8	Navy	No	No	No	No
8207	70	M	CAUC	PTSD	10.45	L10	R7	Army	Yes	Yes	No	No
8827	71	M	CAUC	PTSD	23.5	L8	R9	Marines	Yes	Yes	Unknown	Unknown
8484	74	M	CAUC	PTSD	36.5	R8	L9	Army	Yes	Yes	Unknown	MDD hx
8198	73	M	CAUC	PTSD	5.5	R8	L8	Army	Yes	Unknown	No	No
6893	71	M	CAUC	PTSD	36.5	L9	R9	Unknown	Unknown	Unknown	Unknown	Unknown

**Table 2:** Medications and cause of death.

ID	Age	Primary dx	Medications at Death	Cause of death
8708	71	Control	Insulin, blood thinner, meds for hypertension, hyperlipidemia, thyroid	Coronary Artery Disease & Diabetes Mellitus
8099	74	Control	Unknown	Multiple Myeloma
8111	70	Control	Brovana, Lipitor, Lasix, hydralazine, metoprolol, xarelto, budesonide, zyprexa, Ferrous sulfate, crestor, zofran, albuterol, ferrlecit, perforomist, retacrit, cyanocabalamin, methyprednisone, lovenox, isosobrid, iron, B12, potassium, predinsone, CPAP, hemodialysis	Unknown- but hospitalized due to congestive heart failure, pleural effusions and kidney failure
9974	72	Control	Metoprolol- in ER: ativan, lasix, nitrobid, lidocaine, amiodarone, atropine, epinephrine, bicarb, nitroglycerin, furosemide	Er dx at TOD: bilateral pulmonary edema, congestive heart failure, sustained ventricular tachycardia
7497	73	Control	Lisinopril, carvedilol, atorvastatin, D3, DC, ezetimibe, zinc. ER: epinephrine, naloxone, NA bicarb, calcium, Magnesium	COD pending per narrative, recent covid (still had cough), hx hypertension
8207	70	PTSD	Unknown	Unknown
8827	71	PTSD	Unknown	Unknown
8484	74	PTSD	sertaline	Metastatic melanoma
8198	73	PTSD	Unknown	Small cell lung cancer
6893	71	PTSD	Unknown	Unknown

**Table 3:** Antibodies.

Antibody	Part #	Heat/No Heat	Dilution Factor
Thr231	AA4600032	Heat	1:10
AT8	MN1020	Heat	1:10
Iba1	01919741	Heat	1:50
GFAP	#3670	Heat	1:10
CX3CR1	14609381	No heat	1:10
S100Beta	109250319	No heat	1:10
CD68	E307V	No heat	1:10
vGlut	AB1502991001	No heat	1:10
vGat	CL2793/MA524643	heat	1:10
NeuN	ab177487	heat	1:10
Abeta	BioLegend803001	no heat	1:20
NMDAR (1)	32–0500	heat	1:10
NMDAR 2a	AB1555P	no heat	1:50
NMDAR 2b	ab93610	No Heat	1:50
AMPAR1	13185	No heat	1:10
GABA-B R2	MAB104561	No heat	1:25
GABA Transporter 2	ab229815	Heat	1:10
GABA- B R1	3835	no heat	1:10
anti-mGluR5	MABN540	no heat	1:10
anti-mGluR2/3	06–676	no heat	1:10
mglur 7	ab85343	no heat	1:10
GAD67	AF2086	No heat	1:200
Vimentin	MA5–11883	no heat	1:20

**Table 4:** Statistics for differentially expressed genes.

Gene ID	Fold Change	t-statistic	df	p-value	Cohen’s d
PSEN1	2.066	4.637	7	0.0012	3.111
MAPT	2.194	3.39	8	0.0047	2.144
APP	2.662	2.057	7	0.0394	1.38
NCSTN	1.681	2.642	6	0.0192	1.868
APOE	6.72	3.217	6	0.0235	2.275
ABCA1	4.285	2.898	7	0.0139	1.944
CLU	2.352	3.356	7	0.0061	2.251
A2M	1.485	3.903	6	0.004	2.76
CTSC	3.122	4.025	6	0.0083	2.846
CTSD	2.347	3.03	7	0.0096	2.032
SERPINA3	2.74	2.064	8	0.0364	1.305
CASP4	6.459	1.907	7	0.0459	1.279
GNB2	2.474	11.296	7	<0.001	7.578
GNB1	4.047	3.925	7	0.0044	2.633
PRKCA	1.853	2.016	8	0.0392	1.275
PRKCZ	1.456	2.108	6	0.0398	1.49
PRKCG	1.614	1.997	7	0.0423	1.34
APBA1	1.818	1.706	6	0.0409	1.246
NTRK2	1.999	1.938	8	0.0443	1.226
HSD17B10	2.9	−2.735	7	0.0146	1.835
UQCRC2	3.507	−2.065	7	0.0349	1.385
MPO*	0.331	−2.001	8	0.0506	−1.266
NTRK1*	0.467	−1.922	6	0.0515	−1.359

## Data Availability

All data supporting the findings of this study are available within the paper and its Supplementary Information. PCA Loadings are provided in Supplementary Table 3. Data and stats for all nodes and edges are provided in Supplementary Tables 3 and 4, respectively. Raw data are provided upon request, and complete statistical results are available in Supplementary Tables 1 and 4.

## Highlights

- PTSD is associated with Alzheimer’s disease–relevant molecular remodeling in the human amygdala
- Amyloid burden, pTau231, and markers of gliosis are elevated in the amygdala of older adults with PTSD
- Transcriptional profiling reveals coordinated upregulation of inflammatory, lipid, and proteostatic pathways
- Gene network analysis identifies an APP-centered molecular architecture in PTSD amygdala
- Synaptic markers indicate increased excitatory signaling and reduced inhibitory regulation
- Findings provide human evidence linking trauma-related neuroimmune processes to neurodegenerative vulnerability

## Abbreviations

PTSD: Posttraumatic Stress Disorder
AD: Alzheimer’s disease
ADRD: Alzheimer’s disease and Related Dementia
